# Safety of stereotactic body radiation therapy for localized prostate cancer without treatment planning MRI

**DOI:** 10.1186/s13014-022-02026-1

**Published:** 2022-04-02

**Authors:** Katherine Amarell, Anna Jaysing, Christopher Mendez, Jonathan A. Haas, Seth R. Blacksburg, Aaron E. Katz, Astrid Sanchez, Angela Tong, Todd Carpenter, Matthew Witten, Sean P. Collins, Jonathan W. Lischalk

**Affiliations:** 1 Department of Radiation Medicine, Georgetown University School of Medicine, Washington D.C., 20007 USA; 2 Department of Radiation Oncology, New York University Long Island School of Medicine, Mineola, MY 11501 USA; 3 Department of Radiation Oncology, Perlmutter Cancer Center at New York University Langone Hospital – Long Island, 150 Amsterdam Ave., New York, NY 10023 USA; 4 Department of Radiation Medicine, Lenox Hill Hospital – Northwell Health, New York, NY 10075 USA; 5 Department of Urology, New York University Long Island School of Medicine, Mineola, NY 11501 USA; 6 Department of Radiology, New York University Grossman School of Medicine, New York, NY 10016 USA; 7 Department of Radiation Medicine, Medstar Georgetown University Hospital, Washington D.C., 20007 USA

**Keywords:** Magnetic resonance imaging, Prostate cancer, Stereotactic body radiation therapy, Quality of life, Toxicity

## Abstract

**Background:**

The use of treatment planning prostate MRI for Stereotactic Body Radiation Therapy (SBRT) is largely a standard, yet not all patients can receive MRI for a variety of clinical reasons. Thus, we aim to investigate the safety of patients who received CT alone based SBRT planning for the definitive treatment of localized prostate cancer.

**Methods:**

Our study analyzed 3410 patients with localized prostate cancer who were treated with SBRT at a single academic institution between 2006 and 2020. Acute and late toxicity was evaluated using the Common Terminology Criteria for Adverse Events version 5.0. Expanded Prostate Cancer Index Composite (EPIC) questionnaires evaluated QOL and PSA nadir was evaluated to detect biochemical failures.

**Results:**

A total of 162 patients (4.75%) received CT alone for treatment planning. The CT alone group was older relative to the MRI group (69.9 vs 67.2, *p* < 0.001) and had higher risk and grade disease (*p* < 0.001). Additionally, the CT group exhibited a trend in larger CTVs (82.56 cc vs 76.90 cc; *p* = 0.055), lower total radiation doses (*p* = 0.048), and more frequent pelvic nodal radiation versus the MRI group (*p* < 0.001). There were only two reported cases of Grade 3 + toxicity within the CT alone group. Quality of life data within the CT alone group revealed declines in urinary and bowel scores at one month with return to baseline at subsequent follow up. Early biochemical failure data at median time of 2.3 years revealed five failures by Phoenix definition.

**Conclusions:**

While clinical differences existed between the MRI and CT alone group, we observed tolerable toxicity profiles in the CT alone cohort, which was further supported by EPIC questionnaire data. The overall clinical outcomes appear comparable in patients unable to receive MRI for their SBRT treatment plan with early clinical follow up.

**Supplementary Information:**

The online version contains supplementary material available at 10.1186/s13014-022-02026-1.

## Background

Stereotactic body radiation therapy (SBRT) has become an established form of curative treatment for localized prostate cancer. The precise dose delivery of SBRT yields a steep dose fall off that minimizes high dose radiation exposure to normal tissues thus allowing for ultra-hypofractionated treatment. As a consequence of exceptionally conformal dosimetry, small changes in target volumes can result in significant changes in radiation dose distribution relative to that seen with larger margin conventional fractionation. Consequently, detriments to oncologic outcomes or undesired toxicities to adjacent organs such as the bladder and rectum may theoretically occur [1]. As a result, it is essential to accurately define and contour the prostate and surrounding anatomy with the most detailed imaging available.

Beginning in the late 1990’s, advances in imaging with prostate MRI allowed for fusion with radiation treatment CT scans in an effort to better define pelvic anatomy [2]. This was a paradigm shift from conventional treatment techniques previously based on two-dimensional imaging. Throughout the 2000’s, evolutions in treatment planning and image guided radiation in concert with these advanced diagnostic imaging techniques eventually allowed for dose escalation in the form of SBRT due in part to the improved definition of the prostate and surrounding structures [2].

In the modern era, the use of magnetic resonance imaging (MRI) for treatment planning has become widely used in standard practice. MRI has been demonstrated to better characterize prostatic anatomy compared to pelvic CT alone and has also been shown to have higher sensitivity for detecting extracapsular involvement of cancers [3]. In fact, it has been documented that prostate volumes can appear 40% larger on CT relative to MRI [4]. As a result, the use of MRI fusion during radiation treatment planning has resulted in statistically smaller clinical target volumes (CTV) relative to treatment plans using CT alone [3, 5]. Moreover, MRI treatment plans have been shown to have lower inter-observer variation in contours than that of CT scans alone [6]. As a result, it is suggested that the use of MRI can result in improved target and OAR contours and thus clinical outcomes [4].

Conflicting literature exists with regards to the tangible clinical results of MRI based treatment planning in prostate cancer. The utilization of CT alone versus CT plus MRI fusion for prostate SBRT was studied in the UK in 2018 and concluded both groups had similar contour size and planning target volume (PTV) coverage [1]. In fact, the authors report CT only contouring resulted in reduced rectal dose versus fused MRI treatment plans, which theoretically could result in favorable long-term toxicity profiles. This study offered the conclusion that MRI fusion may be omitted for treatment planning in prostate SBRT [1]. Other studies focusing on conventionally fractionated radiation therapy in the early 2000’s offer opposing data, demonstrating smaller CTVs and rectal radiation dose with the use of MRI [5, 7, 8]. These studies, however, focused on comparing MRI alone to CT alone in radiation treatment planning, while Henderson et al. studied fused MRI in comparison to CT alone. Regardless of the dose discrepancies, none of these studies offered clinical outcomes to the postulated dose and volume differences.

A consensus regarding the necessity of MRI in SBRT treatment planning has yet to be made. From a practical standpoint, it is not always feasible to obtain an MRI for a variety of reasons including incompatible implanted devices, claustrophobia, or insurance/access issues. As such, it is unclear if lack of treatment planning MRI precludes patients from eligibility for prostate SBRT. This study aims to investigate the short-term clinical outcomes including toxicity, quality of life, and biochemical control of patients who did not receive an MRI as a component of their prostate SBRT treatment plan.

## Materials and methods

### Patient eligibility

This single institution review of patients treated with SBRT for prostate cancer was approved by the local Institutional Review Board (Study # 00001269). All patients were evaluated by a radiation oncologist and deemed appropriate for definitive SBRT. All patients underwent pre-treatment diagnostic tests including clinical examination, PSA, and transrectal ultrasound-guided biopsy. Patients were then categorized into standard NCCN prostate risk group classifications. All patients underwent placement of fiducial markers in the prostate approximately one week prior to radiation simulation. Fiducial markers were utilized for inter- and intra-fractional image guidance using a robotic radiosurgical platform. All patients included in the demographic analysis were determined to have detailed treatment planning information to determine MRI inclusion.

### Simulation, planning, and treatment delivery

All patients underwent computed tomography (CT)-based radiation treatment planning simulation (GE Optima 580). For those patients who underwent an MRI of the prostate at the time of simulation, it was fused with the primary simulation CT scan at the level of the prostate to assist in target volume delineation. Patients who did not have dose/fractionation or radiation treatment planning imaging information available were excluded from dosimetric and radiation characteristic analysis. Patients were recommended enema usage prior to simulation and delivery of each treatment fraction. Target volume contours were generated using previously defined definitions. Nodal radiation was incorporated for those patients deemed to be at high risk of occult nodal involvement. Organs at risk (OAR) were contoured and included rectosigmoid, bladder, penile bulb, small bowel, and femoral heads.

Clinical target volume included the entire prostate and proximal seminal vesicles to their bifurcation. A 5 mm isometric expansion of the CTV was created with a tighter, 3 mm, posterior margin to create the PTV. Dose calculations and planning optimization were performed using Accuray MultiPlan software. All patients were treated using SBRT delivered over 5 treatment fractions (total dose of 3500 or 3625 cGy) or with pelvic nodal radiation delivered to 4500 cGy in 180 cGy fractions followed by a 3 fraction boost to the prostate and proximal seminal vesicles (total boost dose of 1650 to 2100 cGy). Treatments were delivered using a robotic radiosurgical platform with prostate motion accounted for in the x-, y-, and z-plane.

### Follow-up5

Toxicity was reported using the Common Terminology Criteria for Adverse Events (CTACE) version 5.0. Patients were followed using serial PSA and clinical examination commonly at 3 to 6 month intervals. Toxicity was measured from completion of SBRT and was graded retrospectively based on an institutional prostate SBRT database and clinical documentation. Acute toxicity was defined as occurring < 90 days after completion of SBRT with the remainder considered late toxicity. Biochemical progression was defined in accordance with the Phoenix definition that is 2 ng/mL rise above PSA nadir. Patients without follow up PSA were excluded from the oncologic analysis.

Health-related quality of life (HRQOL) was reviewed for patients who underwent Expanded Prostate Cancer Index Composite (EPIC) questionnaires pre- and post-SBRT [9]. First, the multi-item scale scores were transformed linearly to the 0 to 100 scale. Then, HRQOL data for the urinary, bowel, and sexual domains was reviewed, which included domain-specific subscales. Due to variations in follow up schedules, patients who completed post-SBRT EPIC questionnaires were grouped into follow up at 1, 3 to 4, and 6 to 9 months and changes in both baseline summary and subscales were evaluated. In order to determine the clinical relevance of HRQOL changes from baseline, minimally important difference (MID) was utilized and was set at half a standard deviation in keeping with prior publications [10, 11].

### Statistical analysis

Statistical analysis was performed using both the Statistical Package for Social Sciences (SPSS) version 24 (Armonk, NY) and Microsoft Excel. The MRI and CT only group demographics, PSA grading, and treatment data were compared using Pearson Chi-Square test for independence and Student t-tests assuming unequal variances. P-values less than 0.05 were considered statistically significant. Biochemical control was analyzed using the Kaplan Meier method.

## Results

### Patient and tumor characteristics

A total of 3,410 patients were identified with detailed treatment planning information available who received SBRT for prostate cancer between 2006 and 2020. The median year of treatment was 2017 (mean of 2016) for both cohorts, thus a transition in MRI frequency through the years likely did not entirely explain the variability between cohorts. A total of 162 (4.75%) of these patients received CT alone in preparation for SBRT. The most common reasons for MRI omission in this cohort were pacemaker (37.7%), AICD (16.05%), and image fusion difficulty due to differences in CT and MRI anatomy (6.79%). Other less common MRI abstention reasons included implants or metal hardware (5.56%), metal fragments or bullet (4.32%), claustrophobia/anxiety (2.47%), MRI intolerability not otherwise specified (2.47%), patient refusal of MRI (2.47%), neurostimulator devices (1.23%), VP shunts (0.62%), and patient illness (0.062%). The CT-alone patients were found to be on average older at the time of SBRT compared to the MRI cohort (69.9 vs. 67.2 years, *p* < 0.001). Disease characteristics between the two groups also demonstrated differences, with the CT alone cohort found to have significantly higher grade group disease (*p* < 0.001) resulting in notably more high risk group cancer (*p* < 0.001). However, pre-treatment PSA was statistically similar between the two groups (9.7 vs 9.3 ng/mL, *p* = 0.643) as was AJCC 7^th^ edition staging (*p* = 0.279). Finally, there was a significant difference in use of ADT as a component of treatment (*p* = 0.046) consistent with the higher risk of disease identified in the CT alone cohort. Patient and tumor characteristics are listed in Table 1.

**Table 1:** Patient and tumor characteristics

	MRI		CT alone		*p*-value
	Patient number	Average (95% CI)	Patient number	Average (95% CI)	
Age	3248	67.2 (66.9–67.4)	162	69.9 (68.7–71.2)	** < 0.001**
PSA (mg/mL)	3248	9.3 (8.7–9.9)	162	9.7 (8.1–11.2)	0.643

	Patient number	Percentage	Patient number	Percentage	*p*-value
*AJCC 7-edition stage*					
Tx	105	3.23	1	0.62	0.279
T1	2512	77.34	123	75.93	
T2	588	18.10	38	23.46	
T3–T4	43	1.32	0	0	
*Grade group*					
1	862	26.54	38	23.46	** < 0.001**
2	1200	36.95	52	32.10	
3	704	21.67	25	15.43	
4	318	9.79	34	20.99	
5	164	5.05	13	8.02	
*Risk Group*					
Low	670	20.63	29	17.90	** < 0.001**
Intermediate	1993	61.36	81	50.00	
High	585	18.01	52	32.10	

Reason	Patient Number (n = 162)	Percentage			
*Reasons for MRI omission*					
Pacemaker	61	37.65			
AICD	26	16.05			
MRI incompatible implanted device*	19	11.72			
Image fusion not possible due to anatomy	11	6.79			
Claustrophobia, anxiety, or patient refusal	8	4.93			
Intolerability not otherwise specified	37	22.83			

*Implant, hardware, bullets, shrapnel, neurostimulator, VP shunt

### Radiation treatment and dosimetric characteristics

Of the 162 patients with CT alone planning, 160 patients had complete radiation records available for dosimetric analysis. Overall, prostate CTV was found to be statistically insignificant in the CT alone group compared to those who received treatment planning MRI (82.56 vs. 76.90 cc; *p* = 0.055). Moreover, the CT alone cohort was found to receive significantly more nodal irradiation relative to the MRI group (22.50 vs. 13.03%, *p* < 0.001). For those patients who received pelvic nodal radiation, there was no significant difference in the dose/fractionation schedule for the boost portion of treatment (*p* = 0.264). The remainder of patients were treated with prostate and seminal vesicle radiation alone with either 3625 cGy or 3500 cGy in 5 fractions. Though the CT patients exhibited higher risk disease, the difference in dose distribution between the CT and MRI group was statistically significant with 10.77% (n = 349) of patients in the MRI group receiving 3625 cGy compared to only 5.00% (n = 8) in the CT alone group (*p* = 0.048) (Table 2).

**Table 2:** Dosimetric and radiation characteristics

Treatment Plan	MRI		CT alone		*P*-value
	Percentage	Number of patients (3239)	Percentage	Number of patients (160)	
*Prostate and SV only*	Total = 86.97%	Total = 2817	Total = 77.50%	Total = 124	
3625 cGy in 5 fractions	10.77%	349	5.00%	8	**0.048**
3500 cGy in 5 fractions	76.20%	2468	72.50%	116	
Pelvic Nodal Radiation*	Total = 13.03%	Total = 422	Total = 22.50%	Total = 36	
1950 cGy in 3 fractions	2.35%	76	1.25%	2	0.264
2100 cGy in 3 fractions	10.59%	343	21.25%	34	
1800 cGy in 3 fractions	0.06%	2	0%	0	
1650 cGy in 3 fractions	0.03%	1	0%	0	
*Volumes*					
Prostate CTV (cc) (95% CI)	76.90 (75.74–78.06)	2814	82.56 (76.89–88.23)	138	0.055
*ADT*					
Yes	797	24.54%	51	31.48%	**0.046**
No	2451	75.46%	111	68.52%	

*Patients underwent 4500 cGy in 25 daily fractions to the pelvis followed by the aforementioned SBRT boost

### Toxicity outcomes

Treatment related toxicity was evaluated for a total of 156 of the 162 patients within the CT-alone group with a median follow up of 18 months. There were a total of 26 CTCAE toxicity events within 16 patients identified in the CT-alone cohort. The median time to any toxicity was 11 months. The following CTCAE toxicity type distribution was observed: renal and urinary (n = 16; 62%), gastrointestinal (n = 8; 31%), and reproductive system (n = 2; 7%). Thirty-one percent of the observed toxicities were classified as acute and predominately manifested as genitourinary symptoms. Disease risk grouping for those who developed toxicity reflected that of the overall CT alone cohort – low (15%), intermediate (58%), and high (27%). A total of three patients who developed any toxicity were treated with pelvic nodal irradiation, and only a minority of patients who developed any toxicity were treated with concurrent ADT (n = 5).

There was an extremely low rate of high-grade toxicity with only two reported cases of grade 3 + toxicity observed in the entire CT alone cohort. One patient experienced grade 3 hematuria, which occurred 21 months post-SBRT. This patient was diagnosed with high-risk disease and received 3500 cGy in 5 fractions. He subsequently required a cauterization via cystoscopy to control hematuria. A second patient developed grade 3 proctitis at 21 months post-treatment. This patient was diagnosed with intermediate risk disease and received 2100 cGy in 3 fractions following 4500 cGy of conventionally fractionated pelvic nodal radiation. Overall, the most common genitourinary and gastrointestinal toxicities were dysuria (n = 7) and rectal hemorrhage (n = 5), respectively. No medical management was required as they did not exceed grade 1. Toxicity details are listed in Table 3.

**Table 3:** Computed tomography alone CTCAE toxicity

CTCAE Type	Time to toxicity (months)	Risk Group	Total dose (cGy)	IDL (%)	Bladder Dmax (cGy)	Rectal Dmax (cGy)
*Renal and urinary*						
Grade 3 Hematuria	21	High	3500	84	Unavailable	Unavailable
Grade 2 Hematuria	135	High	2100	84	2438	2326
Grade 2 Hematuria	23	Intermediate	3500	85	3793	3792
Grade 2 Urinary Frequency	6	Intermediate	3500	83	Unavailable	Unavailable
Grade 1 Urinary Frequency	15	Low	3500	83	3976	3821
Grade 1 Urinary Frequency	0.7	Intermediate	3500	85	3682	3660
Grade 1 Urinary Urgency	15	Low	3500	83	3976	3821
Grade 1 Urinary Urgency	0.7	Intermediate	3500	85	3682	3660
Grade 1 Urinary Incontinence	1	Low	1950	86	2083	2040
Grade 1 Dysuria	0.6	Intermediate	3500	88.3	3665	3663
Grade 1 Dysuria	6	Intermediate	3500	83.3	3828	3791
Grade 1 Dysuria	0.7	Intermediate	3500	85	3682	3660
Grade 1 Dysuria	15	Low	3500	83	3976	3821
Grade 1 Dysuria	15	Intermediate	3500	83	3878	3875
Grade 1 Dysuria	0.7	Intermediate	3500	84	3859	3868
Grade 1 Dysuria	6	Intermediate	3500	83	Unavailable	Unavailable
*Gastrointestinal*						
Grade 3 Proctitis	21	Intermediate	2100	83	2398	2333
Grade 2 Proctitis	15	Intermediate	3500	83	3878	3875
Grade 1 Diarrhea	0.7	Intermediate	3500	84	3859	3868
Grade 1 Rectal Hemorrhage	0.6	High	2100	85	2222	2210
Grade 1 Rectal Hemorrhage	12	Intermediate	3500	84	Unavailable	Unavailable
Grade 1 Rectal Hemorrhage	16	High	3500	86	3813	3783
Grade 1 Rectal Hemorrhage	11	High	3500	86	3813	3783
Grade 1 Rectal Hemorrhage	4	High	3625	84	Unavailable	Unavailable
*Reproductive system and breast*						
Grade 2 Erectile Dysfunction	11	High	3500	86	3813	3783
Grade 2 Erectile Dysfunction	39	Intermediate	3500	84	3859	3868

No association was identified between toxicity development and available dosimetric parameters within the CT alone cohort. Dosimetric analysis revealed bladder Dmax for those patients who did and did not develop genitourinary toxicity in the CT alone cohort to be 3467 vs. 3454 cGy, *p* = 0.96. Similarly, rectal Dmax for those patients who did and did not develop gastrointestinal toxicity in the CT alone cohort was found to be 3214 vs. 3338 cGy, *p* = 0.77. Of note, 146 patients were included in the dosimetric analysis with 14 patients excluded due to lack of available dosimetric data including five patients within the toxicity group.

### EPIC quality of life outcomes

Patient reported quality of life in the CT alone cohort was assessed with EPIC questionnaires and analyzed prior to treatment and at specific post-SBRT intervals. There was notable attrition for patients who completed EPIC questionnaires, nevertheless over half of the CT alone cohort had necessary information available for analysis. Of the total 162 patients within the CT alone cohort, 99, 90, and 89 patients completed analyzable EPIC questionnaires in the urinary, bowel, and sexual domains, respectively. Baseline mean EPIC domain summary scores were found to be 84 ± 13 (n = 99), 93 ± 10 (n = 90), and 43 ± 29 (n = 89) in the urinary, bowel, and sexual domains, respectively. At one month following treatment, a notable decline in urinary quality of life was observed that met MID, however by 3–4 months this urinary decline had resolved with persistent resolution at 6–9 months. A consistent decline at 1 month with subsequent resolution at 3–4 months was observed for all urinary domain-specific HRQOL subscales.

Baseline EPIC bowel summary scores revealed a very similar clinically meaningful decline at one month with a notable mean drop from baseline of 18. This decline was driven by both domain-specific HRQOL subscales – function and bother. Analogous to the urinary domain, bowel quality of life improved at 3–4 months and no longer registered as a MID for both bowel subscales. However, a statistically significant decline in bowel summary scores was noted at 6–9 months follow up.

Finally, baseline EPIC sexual summary scores revealed low baseline status possibly due to the older age of the cohort. There was a clinically insignificant decline at 1 month (mean difference of -9.1) with improvement at 3–4 months (mean difference of -8.2) and 6–9 months (mean difference of -1.5), though these values did not represent clinically meaningful drops. Domain-specific HRQOL subscales of function and bother revealed similar trends with mild declines followed by improvement in score, though only the bother domain at 3–4 months follow-up met MID. Additional file 1, 2, 3: Figs. S1 to S3 illustrated EPIC HRQOL for urinary, bowel, and sexual domain summary scores. Detailed EPIC HRQOL data including subscale analysis is available in Tables 4, 5 and 6.

**Table 4:** EPIC questionnaire—urinary

	Baseline (n = 99)	1 month (n = 26)	3–4 months (n = 16)	6–9 months (n = 18)
*HRQOL Urinary domain summary*				
Mean	83.53	− 14.13*	0.07	− 2.96
Standard deviation	13.40			
MID	6.70			
*Domain-specific HRQOL subscales*				
Function				
Mean	90.5	− 10.8*	0.5	− 1.1
Standard deviation	12.1			
MID	6.0			
*Bother*				
Mean	79.54	− 17.45*	− 1.19	− 4.94
Standard deviation	17.63			
MID	8.82			
*Incontinence*				
Mean	88.92	− 8.30*	1.44	− 2.07
Standard deviation	16.48			
MID	8.24			
*Irritative/Obstructive*				
Mean	83.43	− 16.14*	0.50	− 4.86
Standard deviation	14.17			
MID	7.08			

*Indicates a clinically significant decline in HRQOL based on MID

**Table 5:** EPIC questionnaire—bowel

	Baseline (n = 90)	1 month (n = 26)	3–4 months (n = 14)	6–9 months (n = 17)
*HRQOL Bowel domain summary*				
Mean	92.76	− 18.03*	0.86	− 5.89*
Standard deviation	9.52			
MID	4.76			
*Domain-specific HRQOL subscales*				
Function				
Mean	91.92	− 17.88*	2.98	− 4.95
Standard deviation	10.95			
MID	5.47			
*Bother*				
Mean	94.32	− 18.18*	− 1.97	− 4.27
Standard deviation	10.34			
MID	5.17			

*Indicates a clinically significant decline in HRQOL based on MID

**Table 6:** EPIC questionnaire—sexual

	Baseline (n = 89)	1 month (n = 26)	3–4 months (n = 13)	6–9 months (n = 13)
*HRQOL Sexual domain summary*				
Mean	42.98	− 9.10	− 8.20	− 1.51
Standard deviation	28.85			
MID	14.43			
*Domain-specific HRQOL subscales*				
Function				
Mean	39.32	− 7.92	− 5.89	− 4.85
Standard deviation	29.89			
MID	14.95			
*Bother*				
Mean	56.16	− 2.21	− 24.22*	5.77
Standard deviation	37.87			
MID	18.94			

*Indicates a clinically significant decline in HRQOL based on MID

### Oncologic outcomes

**Fig. 1:**
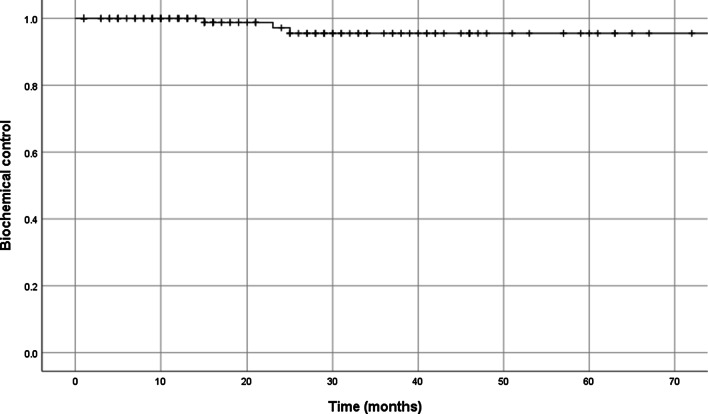
Kaplan Meier curve of biochemical control in CT alone cohort

A total of 127 patients within the CT alone cohort had available post-treatment PSA with very early follow-up for analysis. At a median follow up of 21 months, we identified a total of five Phoenix definition biochemical failures. No radiological alone failures were identified in this cohort. The risk group distribution for those who failed was as follows: low (n = 0), intermediate (n = 3), and high (n = 2). Median time to failure was 25 months. None of these patients were treated with nodal irradiation nor did any failures also occur in patients who developed CTCAE toxicity. Figure 1 illustrates the Kaplan Meier for biochemical control in the CT alone cohort.

## Discussion

Magnetic resonance imaging is currently standard in the treatment planning of SBRT for localized prostate cancer. However, many patients are unable to obtain a prostate MRI. Common contraindications are associated with heart conditions (e.g. presence of cardiac implantable devices) as well as other conditions commonly seen in the elderly population including dental implants, vascular clips, catheters, neurostimulators and hearing aids [12]. While these are only a few of the absolute contraindications to MRI, many other relative contraindications exist such as certain arterial stents, joint prosthesis, penile prosthesis, and IVC filters [12]. In examining pacemakers alone, the scope of these limitations can be appreciated. The average age at prostate cancer diagnosis is 66 years and the prevalence of individuals aged 64 to 74 years with an implanted pacemaker is more than 1 in 160 [13, 14]. Furthermore, up to 14% of patients who undergo MRI experience significant claustrophobia [15]. Even if a patient with claustrophobia is able to undergo an “open” MRI, the quality of their scan can be degraded by excessive movement due to anxiety. Hence, there are numerous barriers that may prevent patients from obtaining a quality radiation treatment planning MRI. With respect to our cohort, we found the most common reason for omission of MRI to be cardiac devices (pacemaker in 37.65% and AICD in 16.05%). Second to this reason, fusion difficulty due to anatomy was an apparent impediment (6.79%). This study aimed to investigate the safety of CT alone treatment planning for prostate SBRT by evaluating toxicity, patient reported quality of life, and oncologic outcomes in patients treated sans MRIs. To our knowledge, this is one of the first studies to evaluate clinical follow up in patients who did not receive an MRI as a part of their SBRT treatment plan.

We identified important differences between CT alone and MRI cohorts, notably from a demographic standpoint, with those in the CT alone cohort significantly older (69.9 vs. 67.2 years). Although impossible to say with certainly, many of the aforementioned comorbidities precluding the ability to obtain an MRI can increase with age, which could explain this difference. It is also important to note that grade group and risk group were notably more aggressive in the CT alone cohort (*p* < 0.05). This resulted in a significant increase in the use of pelvic nodal irradiation within this group (22.50% vs. 13.03%, *p* < 0.05). Anecdotally, prostate MRI can give the radiation oncologist more confidence that occult pelvic nodal disease is absent and thus allow for deferral of pelvic nodal irradiation for certain subgroups. Within the group of patients who received prostate and seminal vesicle radiation only, the MRI group was associated with a higher total radiation dose (3625 cGy in 5 fractions). It is not uncommon for SBRT radiation dose to be slightly de-escalated (i.e. 3500 cGy in 5 fractions) in the absence of a prostate MRI due to lack of precise anatomical definition, particularly at the prostate-rectal interface. This may be why 3625 cGy has become more ubiquitous in the modern era in concert with the more widespread use of prostate MRI.

From a treatment planning standpoint, we found prostate CTV was not statistically significant in the MRI group than in the CT alone group (*p* = 0.055) with an average difference of approximately 5.6 cc. Though this average difference was found to be insignificant, it is of note that the confidence interval for the CT alone group is relatively wide (± 5.67 cc) compared to that of the MRI group (± 1.16 cc) which likely contributes to the insignificant p-value despite the observed difference. An appreciable difference is outlined elsewhere in literature in which MRI contours were an average of 8.7 cc smaller than that seen with CT alone contours [5]. Areas of contouring that offer the most discrepancy include the posterior prostate as well as the prostatic apex [5]. Additionally, portions of the neurovascular bundles are more commonly included on CT alone contours, which also contributes to larger target volumes [5]. Though previous studies postulate theoretical reasons for toxicity secondary to over-contouring, they failed to investigate clinical outcomes of patients with CT alone treatment planning. Rather, previous studies such as Roach et al. and Henderson et al. utilized the same patient group in order to compare theoretical CT alone and MRI treatment plans.

Magnetic resonance imaging contouring offers clear superiority in delineating prostatic tissue. This is particularly true in the region of fibromuscular stroma and at the prostatic apex, which has been historically poorly defined on CT alone [3]. In addition, the apical portion of the prostate is known to be involved in 75–85% of cancers [5]. Thus, adequate contouring of this region is crucial. On the other hand, inappropriate contouring of the apex could theoretically result in unwanted toxicities such as erectile and genitourinary dysfunction. As such, the therapeutic window is narrower, from an anatomical standpoint, at the apex of the prostate where the target structure is more challenging to identify and if over contoured can result in excess dose to the penile bulb, membranous urethra, and adjacent rectum. Despite the ability of an MRI to identify detailed anatomical information and transfer that information onto the planning CT scan, there are inherent limitations to the transfer process itself. For example, when fusing the CT and MRI, geometric mismatching of the prostate can occur, especially when scans are taken on different days [16]. Regardless, in our CT alone cohort despite the larger CTVs observed, we identified overall very low rates of toxicity and good HRQOL outcomes.

Overall, our study reveals a safe toxicity profile, as the majority of toxicities were CTCAE grade 1 or 2 with only two grade 3 toxicities observed. Of the grade 1 and 2 toxicities, most fell under urinary toxicity, ranging from hematuria (2), urinary frequency (3), urinary urgency (2), urinary incontinence (1), and dysuria (7). The remaining grade 1 and 2 toxicities were associated with gastrointestinal (GI) toxicities in the form of diarrhea (1), proctitis (1), and rectal hemorrhage (5). Of the 16 cases of urinary toxicity, 14 were treated with 3500 cGy in 5 fractions without pelvic nodal radiation. The bladder Dmax for these associated toxicities ranged from 2083 to 3976 cGy. This range indicates a safe radiation profile with acceptable maximum dose delivered to the bladder similar to other clinical trials [17]. Of the 8 cases of GI toxicity, 6 did not receive pelvic nodal radiation. Rectal Dmax for these 8 cases ranged from 2210 to 3875 cGy. Of the 8 cases of GI toxicity, 4 patients were above this constraint, though comparison to other studies necessitates verifying whether absolute maximum or the TG101 specified volumetric maximum of 0.035 cc is utilized. Thus, limiting the Dmax to the rectum in these cases may have prevented acute GI related toxicities [17]. Overall, other institutional studies investigating SBRT with fused MRI treatment plans have shown similar rates or even higher rates of grade 2 + urinary and rectal toxicity [18–20]. King et al. cited acute grade 3 + toxicity at a rate of 1–3% across multiple studies [21]. While our genitourinary toxicity rates was within this range, we acknowledge that a shorter median follow-up of 18 months could impact these results.

Oncologic outcomes using CT alone planning for prostate SBRT can also be a theoretical concern. Without associated MRI imaging, as previously mentioned, detailed identification of prostatic tissue can be challenging. Moreover, prostate MRI adds supplemental information regarding location of PI-RADS lesions, extracapsular extension, and seminal vesicle invasion all of which is taken into consideration during radiation contouring and planning [3]. As such, it is possible that loss of this information could lead to worsened oncologic outcomes when tighter SBRT margins are utilized. Nevertheless, systemic over-contouring, which could be endemic in CT alone planning, may washout this difference. In our cohort we did not see an increased rate of Phoenix definition failures with only five identified after nearly 2 years median follow-up. Though this follow-up duration is quite limited given the natural history of prostate cancer, a significant portion of these patients exhibited high risk disease, making them more susceptible to early failure. Despite this, we found only five patients with early failure in this more aggressive cohort. Nevertheless, longer follow-up is needed to determine if late failures due to the aforementioned concerns may occur.

Patient reported quality of life data was extracted via EPIC questionnaires and evaluated in a large proportion of patients who received CT-alone treatment plans, though there was significant attrition on follow-up questionnaires. Our data replicated prior SBRT prostate publications where MRIs were utilized, which demonstrated a transient flare in gastrointestinal and genitourinary symptoms followed by recovery at later time points [10, 21]. In contrast, we did not identify a gradual decline in sexual function that has been previously published, which may be a consequence of our limited patient numbers as well as low sexual function scores at baseline [10]. Overall, EPIC quality of life questionnaires for patients with CT alone treatment plans exhibited trends similar to that of patients reported in literature who were treated with SBRT using the standard fused MRI in treatment planning [10, 21].

Limitations of the present study include its retrospective nature and relatively short term follow up given the disease site. The data would be further strengthened with prospective analyses such the MIRAGE trial that explores the outcomes of MRI planning alone with MRI-linac platform delivery of prostate SBRT [22]. Our large sample size in comparison with other studies does provide strength in the validity of the outcome. It is important to stress: the authors of this study are not advocating for discontinuing the use of treatment planning MRI for prostate SBRT. However, we recognize in certain clinical situations an MRI may not be feasible. In our CT alone cohort, we found this group of patients to be significantly older, have more aggressive histology, and be more commonly diagnosed with high risk disease. Nonetheless, a dose reduction to 3500 cGy in 5 fractions was more commonly observed in the CT alone cohort, though in contrast elective pelvic nodal irradiation was more frequently employed. Based on our database analysis of CT alone SBRT treatment for prostate cancer, we have observed the following clinical outcomes: (1) very low rates of high-grade toxicity, (2) genitourinary and gastrointestinal quality of life outcomes that mimic that seen when MRIs are utilized, and (3) similar early biochemical control to that seen with use of fusion MRI.

## Conclusion

Magnetic resonance imaging is commonly utilized as a component of radiation treatment planning for prostate SBRT. However, many patients cannot receive prostate MRI for a variety of reasons. We reviewed a large institutional database of patients (n = 3410) treated for localized prostate cancer with SBRT and identified 162 who were treated without a planning MRI. This cohort tended to be older with more aggressive histology. Larger CTVs, lower total radiation doses, and more pelvic nodal irradiation was identified within the CT alone cohort relative to the MRI cohort. Only two grade 3 toxicities were observed within the CT alone cohort. Genitourinary and gastrointestinal patient reported quality of life mimicked that seen in publications where MRIs were utilized. Finally, early biochemical control appeared to be excellent. For those patients unable to receive a planning MRI for prostate SBRT, clinical outcomes appear to be similar when CT alone is utilized for treatment planning.

## Supplementary Information

**Additional file 1: Figure S1.** EPIC urinary summary domain scores (mean) at baseline and post-SBRT. Dashed lines indicate minimally important difference (MID) upper and lower boundaries based on baseline summary score ½ standard deviation.

**Additional file 2: Figure S2**. EPIC Bowel summary domain scores (mean) at baseline and post-SBRT. Dashed lines indicate minimally important difference (MID) upper and lower boundaries based on baseline summary score ½ standard deviation.

**Additional file 3: Figure S3**. EPIC sexual summary domain scores (mean) at baseline and post-SBRT. Dashed lines indicate minimally important difference (MID) upper and lower boundaries based on baseline summary score ½ standard deviation.

## Data Availability

The datasets used and/or analyzed during the current study are available from the corresponding author on reasonable request.

## Abbreviations

SBRT: Stereotactic body radiation therapy
MRI: Magnetic resonance imaging
CTV: Clinical target volume
CT: Computed tomography
OAR: Organs at risk
NCCN: National Comprehensive Cancer Network
CTCAE: Common Terminology Criteria for Adverse Events
HRQOL: Health-related Quality of Life
MID: Minimally important difference
SPSS: Statistical Package for Social Sciences
PSA: Prostate specific antigen
AICD: Automatic implantable cardioverter-defibrillator
AJCC: American Joint Committee on Cancer
ADT: Androgen deprivation therapy
CC: Cubic centimeter
cGy: Centigray
IVC: Inferior Vena Cava
GI: Gastrointestinal
Dmax: Dose max
PI-RADS: Prostate Imaging Reporting and Data System
