# Supplementary material for: Safety of stereotactic body radiation therapy for localized prostate cancer without treatment planning MRI
Source: Radiat Oncol. 2022 Apr 2;17:66. doi: 10.1186/s13014-022-02026-1 (PMC8977039; doi:10.1186/s13014-022-02026-1)
Supplement: Supplementary file 1 — Additional file 1: Figure S1. EPIC urinary summary domain scores (mean) at baseline and post-SBRT. Dashed lines indicate minimally important difference (MID) upper and lower boundaries based on baseline summary score ½ standard deviation. [file 13014_2022_2026_MOESM1_ESM.docx]

**Supplementary Figure 1:** EPIC urinary summary domain scores (mean) at baseline and post-SBRT. Dashed lines indicate minimally important difference (MID) upper and lower boundaries based on baseline summary score ½ standard deviation.
